# A systematic analysis of splicing variants identifies new diagnoses in the 100,000 Genomes Project

**DOI:** 10.1186/s13073-022-01087-x

**Published:** 2022-07-26

**Authors:** Alexander J. M. Blakes, Htoo A. Wai, Ian Davies, Hassan E. Moledina, April Ruiz, Tessy Thomas, David Bunyan, N. Simon Thomas, Christine P. Burren, Lynn Greenhalgh, Melissa Lees, Amanda Pichini, Sarah F. Smithson, Ana Lisa Taylor Tavares, Peter O’Donovan, Andrew G. L. Douglas, Genomics England Research Consortium, Splicing and Disease Working Group, Nicola Whiffin, Diana Baralle, Jenny Lord

**Affiliations:** 1 Faculty of Medicine, Human Development and Health, University of Southampton, Southampton, UK; 2 Faculty of Medicine, National Heart and Lung Institute, Imperial College London, London, UK; 3 Cancer Sciences, Faculty of Medicine, University of Southampton, Southampton, UK; 4 Wessex Clinical Genetics Service, Princess Anne Hospital, Southampton, UK; 5 Wessex Regional Genetics Laboratory, Salisbury District Hospital, Salisbury, UK; 6 Faculty of Medicine, University of Southampton, Southampton, UK; 7 Department of Paediatric Endocrinology and Diabetes, University Hospitals Bristol and Weston NHS Foundation Trust, Bristol, UK; 8 Bristol Medical School, Department of Translational Health Sciences, University of Bristol, Bristol, UK; 9 Liverpool Centre for Genomic Medicine, Crown Street, Liverpool, UK; 10 North East Thames Regional Genomics Service, Great Ormond Street Hospital, London, UK; 11 Department of Clinical Genetics, University Hospitals Bristol and Weston Foundation Trust, Bristol, UK; 12 Genomics England, Dawson Hall, Charterhouse Square, London, UK; 13 Cambridge University Hospitals NHS Foundation Trust, Cambridge Biomedical Campus, Hills Road, Cambridge, UK; 14 Oxford Centre for Genomic Medicine, Oxford University Hospitals NHS Foundation Trust, Oxford, UK; 15 Wellcome Centre for Human Genetics, University of Oxford, Oxford, UK

## Abstract

**Background:**

Genomic variants which disrupt splicing are a major cause of rare genetic diseases. However, variants which lie outside of the canonical splice sites are difficult to interpret clinically. Improving the clinical interpretation of non-canonical splicing variants offers a major opportunity to uplift diagnostic yields from whole genome sequencing data.

**Methods:**

Here, we examine the landscape of splicing variants in whole-genome sequencing data from 38,688 individuals in the 100,000 Genomes Project and assess the contribution of non-canonical splicing variants to rare genetic diseases. We use a variant-level constraint metric (the mutability-adjusted proportion of singletons) to identify constrained functional variant classes near exon–intron junctions and at putative splicing branchpoints. To identify new diagnoses for individuals with unsolved rare diseases in the 100,000 Genomes Project, we identified individuals with de novo single-nucleotide variants near exon–intron boundaries and at putative splicing branchpoints in known disease genes. We identified candidate diagnostic variants through manual phenotype matching and confirmed new molecular diagnoses through clinical variant interpretation and functional RNA studies.

**Results:**

We show that near-splice positions and splicing branchpoints are highly constrained by purifying selection and harbour potentially damaging non-coding variants which are amenable to systematic analysis in sequencing data. From 258 de novo splicing variants in known rare disease genes, we identify 35 new likely diagnoses in probands with an unsolved rare disease. To date, we have confirmed a new diagnosis for six individuals, including four in whom RNA studies were performed.

**Conclusions:**

Overall, we demonstrate the clinical value of examining non-canonical splicing variants in individuals with unsolved rare diseases.

**Supplementary Information:**

The online version contains supplementary material available at 10.1186/s13073-022-01087-x.

## Background

Improved diagnosis of rare genetic diseases remains a significant clinical and research challenge [1]. Diagnostic yields in individuals with rare diseases remain below 50%, despite extensive investigations including whole-genome sequencing [2]. The accurate interpretation of genomic variants in existing sequencing data presents an important opportunity to narrow the diagnostic gap [3].

Splicing is the process by which introns are removed from a pre-mRNA primary transcript. Almost all human protein-coding genes are spliced, and disruption of splicing is a major cause of rare genetic diseases [4]. The improved interpretation of splicing variants is therefore a major opportunity to improve clinical outcomes for individuals with undiagnosed rare disease [5].

Already, “canonical splice site” (CSS) variants within 2 bp of an exon–intron junction are widely annotated as “loss of function” (LoF) variants and are known to be strong diagnostic candidates in “loss of function” disorders [6]. The contribution of non-canonical splicing variants to rare diseases is also becoming increasingly recognised [7]. Up to 27% of pathogenic de novo splicing variants in exome-sequencing data are found in non-canonical positions [8]. Several studies [7–9] have developed the concept of a “near-splice” region, usually tens of base pairs around an exon–intron junction, which contains many conserved splicing motifs.

However, near-splice variants are under-reported in clinical databases [8], and no standards exist for their interpretation. Furthermore, variants distal to the near-splice region, including putative branchpoint variants and deep intronic variants (further than 100 bp from an exon–intron junction [10]), can also disrupt splicing, and their overall contribution to rare diseases is unknown. Individual instances of pathogenic branchpoint variants have been previously described [11, 12], but they have not been systematically characterised in a large rare disease cohort.

Recently, large population genomic datasets have provided the statistical power necessary to measure constraints on genetic variation within human populations. One powerful metric which uses this approach is the mutability-adjusted proportion of singletons (MAPS) [13], which identifies classes of variation which are subject to purifying selection, and are therefore likely to be deleterious. MAPS has previously been calculated in many contexts, including for near-splice positions in the Exome Aggregation Consortium (ExAC) [8], and for upstream start-codon-creating variants in Genome Aggregation Database (gnomAD) [14].

Recent advances in computation and artificial intelligence have led to the development of numerous in silico predictors for the prioritisation of splicing variants [15]. For example, SpliceAI is a machine learning tool which robustly predicts splice sites and splice-disrupting variants [16] and outperforms other algorithms in predicting splicing consequences from sequence data [17]. However, in clinical variant interpretation, well-validated functional assays have greater weight than in silico predictions of variant effect [6], and functional validation of most predicted splice-disrupting variants is still required to confirm a molecular diagnosis.

Here, we perform a systematic analysis of potential splicing variants in whole-genome sequencing data from 38,688 individuals in the Rare Disease arm of the 100,000 Genomes Project (100KGP) [18]. We evaluate the contribution of variants at or near canonical splice sites, and at predicted branchpoints, to rare genetic diseases in this cohort. We show that predicted splicing branchpoints harbour deleterious non-coding variants which are amenable to systematic analysis in WGS data. We used a gene-agnostic approach to prioritise 258 de novo variants which potentially disrupt splicing in families affected by a rare genetic disorder. Of these, at least 84 were already considered to be diagnostic, and we identified an additional 35 variants which are likely to be diagnostic given the available molecular, phenotypic, and in silico data. We confirmed a new molecular diagnosis for six participants, including four out of five participants for whom RNA studies were performed. Ultimately, we demonstrate the clinical and diagnostic value of examining both canonical and non-canonical splicing variants in unsolved rare diseases.

## Methods

### Cohort, sequencing, and tiering

This analysis was performed on whole-genome sequencing data from 38,688 participants in the Rare Disease arm of the 100,000 Genomes Project [19]. These comprised 26,660 unaffected parents of rare disease probands, and 12,028 participants (offspring) for whom trio WGS data was available. Only participants for whom WGS data was aligned to GRCh38 were included in this study. Parents affected by a rare genetic disease were excluded from the analysis of variant constraint (see below). Otherwise, participants were not selected or stratified by any other criterion. The sequencing and bioinformatic pipelines, as well as the “tiering” framework for variant prioritisation, have been previously described [18]. Briefly, variants meeting filtering criteria and falling within applied virtual gene panels were annotated as tier 1 (loss of function or de novo protein-altering variants), tier 2 (other variant types, e.g. missense, with correct mode of inheritance), or tier 3 (all other filtered variants). For example, CSS variants in an appropriate gene panel are annotated as tier 1.

### Defining coding sequences and near-splice positions

The code used to perform this and subsequent analyses are available online [20] (see the “Availability of data and materials” section).

We identified coding sequences (CDS) in high-confidence protein-coding transcripts from the GENCODE v29 annotation [21] (GRCh38, Content = "Comprehensive gene annotation", Region = "CHR") using the following filtering criteria: feature type = "CDS", gene_type = "protein_coding", transcript_type = "protein_coding", level ! = "level 3", and tag = "CCDS", "appris_principal_1", "appris_candidate_longest", "appris_candidate", or "exp_conf". 401,314 CDS features (207,548 unique) met these criteria. Only autosomal CDS were included in the subsequent analyses. UTR and other non-coding exons were not included in this analysis.

For each CDS feature, we annotated individual genomic positions with their positions relative to a splice donor or acceptor site, excluding any sites with conflicting annotations. We defined the near-splice region around the acceptor site as 25 bp of intronic sequence (acceptor − 25 to acceptor − 1) and 11 bp of exonic sequence (acceptor 0 to acceptor + 10). Around the donor site, we included 11 bp of exonic sequence (donor − 10 to donor 0) and 10 bp of intronic sequence (donor + 1 to donor + 10). A total of 9,588,491 distinct near-splice positions were identified.

### phyloP

We annotated each near-splice position with phyloP scores from multiple alignments of 99 vertebrate species to the human genome (phyloP 100-way) [22] with pyBigWig, an open-source Python package [23], using BigWig files downloaded from the UCSC Genome Browser (hg38) [24, 25].

### SpliceAI

For every possible near-splice SNV in our positions of interest (i.e. all three possible single base changes at each of the 9,588,491 positions), we annotated the predicted effect on splicing with SpliceAI [16]. We annotated variants with pre-computed genome-wide SpliceAI v1.3 scores (distance parameter = 50 bp, “masked” data, available via https://github.com/Illumina/SpliceAI) using BCFtools v1.9 [26]. A SpliceAI annotation was available for 28,265,193 variants (98.2% of 28,765,473 possible variants).

Aggregate SpliceAI scores for each near-splice position were calculated as the mean probability that any variant at this position disrupts splicing. The probability that a given variant disrupts splicing was calculated as the probability (*P*) of any one of the SpliceAI-predicted splicing events occurring, (i.e. 1-probability of *no* events occurring). SpliceAI gives the probabilities of individual splicing events as a delta score (DS) for each of acceptor gain (AG), acceptor loss (AL), donor gain (DG), or donor loss (DL), giving:

$$P=1-((1-(DS\_AG))\ast(1-(DS\_AL))\ast(1-(DS\_DG))\ast(1-(DS\_DL))$$

### Mutability-adjusted proportion of singletons

In addition to the near-splice SNVs identified above, we also determined the set of all possible coding SNVs in our exons of interest. These were annotated with the reference base for each position (GRCh38, GenBank assembly accession GCA_000001405.15) and its immediate sequence context (1 bp either side) with bedtools version 2.27.1 [27].

We annotated every possible coding SNV within our exons of interest with the Variant Effect Predictor (VEP) version 99 [28]. In order to assign one unambiguous annotation to each variant, only the consequence in one transcript (typically the canonical transcript, as determined by VEP’s “–pick” flag) was used. Only synonymous, missense, and nonsense variants were included in the subsequent analysis. Synonymous variants within a near-splice region were classed as near-splice variants for the MAPS calculation and were excluded from the synonymous variant set. Missense variants within a near-splice region were excluded from the analysis altogether. Nonsense variants within a near-splice region were classed as nonsense variants and excluded from the near-splice variant set.

We interrogated whole-genome sequencing data from 26,660 unaffected parents in the Genomics England (GEL) Rare Disease cohort for SNVs overlapping the near-splice and coding positions defined above using BCFtools v1.9 [26]. Only variants passing all filters (see https://research-help.genomicsengland.co.uk/display/GERE/aggV2+Details) within the GEL aggregated multi-sample VCF were included. We identified 915,024 synonymous, 1,965,441 missense, 53,825 nonsense, and 672,528 near-splice variants and calculated allele counts across the 26,660 unaffected parents for each variant.

We calculated MAPS with custom Python scripts, adapting code written by Short et al. [29] (https://github.com/pjshort/dddMAPS). We used the mutation rate of a given trinucleotide context calculated by Samocha et al. [30]. The proportion of singletons for each position was adjusted for the mutability of the immediate sequence context using a linear model trained on synonymous variants within the same exons. As in previous studies [8, 13], the set of synonymous variants used for the MAPS model was not filtered by any other criterion (e.g. SpliceAI score). Our data are therefore directly comparable with these previous studies. Unselected synonymous variants are a useful baseline for comparison because they capture potential unknown impacts of coding variation, including impacts on splicing or translation efficiency. This is also a more conservative approach, because the inclusion of potentially functional variants in the synonymous baseline would only weaken any apparent constraint signals in other functional variant classes.

### Branchpoints

Splicing branchpoint positions were identified by LaBranchoR, a machine-learning tool trained on experimentally validated branchpoints, which accurately identifies at least one branchpoint for the majority of introns genome-wide [31]. Although several branchpoint prediction tools are available [32], we favoured LaBranchoR because it accurately predicts branchpoints at well-annotated 3′ splice sites, which were the main focus of this analysis. Pre-computed LaBranchoR scores are publicly available for every position 1–70 bp upstream of a splice acceptor (GENCODE v19, hg19) either for download or through the UCSC Genome Browser [31]. For each intron, the highest scoring position was annotated as the branchpoint (BP), totalling 195,863 putative branchpoints.

We converted each branchpoint to hg38 coordinates using the UCSC Liftover tool [24]. We annotated five positions upstream (− 5 to − 1) and five positions downstream (+ 1 to + 5) of each branchpoint, as well as every possible SNV at each of these positions using custom Python scripts. phyloP scores for each position, and SpliceAI scores for each variant, were determined as above. We calculated the MAPS statistic for these branchpoint positions in the same cohort of participants as described above. Comparison of MAPS scores in branchpoint positions was made to the same set of coding variants as described above.

### De novo variants

De novo variants (DNVs) overlapping near-splice positions were identified from a set of 1,004,599 high confidence de novo calls in 13,949 trios from 12,609 rare disease families. The annotation pipeline used to identify these variants is publicly available [33]. Briefly, a multisample VCF for each trio was annotated for putative DNVs using custom scripts. Putative DNVs were then filtered by a series of “Global”, “Base”, and “Stringent” filters (see reference [33]). Unless otherwise stated, our analyses were performed on DNVs aligned to GRCh38 (870,559 DNVs in 12,028 trios).

At the outset of this project this dataset was not available. Preliminary work to identify candidate diagnostic de novo variants was undertaken in a smaller set of 402,464 variants identified through a custom filtering strategy by Patrick J. Short (Wellcome Sanger Institute, personal correspondence). These variants were identified by applying post-processing filters to DNVs in 4967 trios identified by the Platypus variant caller [34] in GEL and aligned to GRCh38. They were filtered according to the following criteria: genotype heterozygous in offspring and homozygous reference in both parents, no more than one alternate allele read in either parent, variant allele frequency in the offspring between 0.3 and 0.7, greater than 20 sequencing reads in the offspring and both parents, fewer than 98 sequencing reads in the offspring, no overlap with locus control regions, no overlap with hg38 “patch regions”, no other DNV within 20 bp in the same individual. Some candidate variants identified in this preliminary dataset are not present in the larger de novo set, owing to differences in the filtering pipeline. Unless explicitly stated, the data presented here are from the larger DNV set, above.

### Candidate diagnostic variants

To identify candidate diagnostic near-splice and branchpoint variants, we annotated all GRCh38 autosomal de novo SNVs passing the “stringent” filters (above) with VEP (version 99). For each variant, to maximise our sensitivity to identify variants in known developmental disorder genes, the consequence in one transcript per gene (determined by VEP’s “–per_gene” flag) was determined. We annotated these variants with SpliceAI as described above, although SpliceAI was not used to prioritise these variants and no SpliceAI score cut-off was applied. We filtered for variants overlapping our branchpoint or near-splice positions of interest (adjacent to coding exons only), finding 3672 such variants. Where a variant had both a branchpoint and a near-splice annotation, only the near-splice annotation was kept. We then filtered for variants overlapping any known monoallelic rare disease gene with a loss of function mechanism using the G2P DD, G2P Eye, and G2P Skin gene lists [35] (accessed 27/10/2021, confirmed and probable genes only). In total, we identified 258 candidate splicing DNVs (238 near-splice, 20 branchpoint) in 255 participants, adjacent to coding exons in 137 genes.

To identify new diagnoses in the cohort, we annotated these variants with tiering data, phenotype data, and participant outcome data from the GEL bioinformatics pipeline [36]. For each participant and DNV, the similarity between the HPO terms recorded at recruitment and the phenotype expected for a loss-of-function variant in that gene from the G2P [35] and OMIM [37] databases were manually compared by a Paediatrician and a Consultant Clinical Geneticist. Plausible phenotype matches were confirmed or refuted through literature search and through detailed clinical review by the recruiting clinician. Excluding any participants whose case was already solved through 100KGP, we identified 35 new likely diagnostic variants with at least a plausible phenotype match. In each instance, we placed a clinical collaboration request with Genomics England to recruit the participant to the Splicing and Disease Study for functional characterisation of the variant.

Genomics England does not allow re-identification of participants outside of a secure research environment. In order to protect participant identities, the HPO terms given here are “abstracted” by moving up one level in the HPO hierarchy. For example “Tetralogy of Fallot” becomes “Conotruncal defect”.

### Functional validation

Samples from five participants underwent functional characterisation through the Splicing and Disease study at The University of Southampton. Blood was collected in PAXgene Blood RNA tubes, with the PAXgene Blood RNA kit (PreAnalytix, Switzerland) used to extract RNA. Random hexamer primers were used to synthesise complementary DNA (cDNA) by reverse transcription using the High-Capacity cDNA Reverse Transcription kit (Thermo Fisher Scientific).

Reverse transcription polymerase chain reaction (RT-PCR) was used to test for splicing alterations. Primers were designed for each variant to include at least two exons up- and downstream of the target (primer sequences available upon request). Agarose gel electrophoresis was used to assess participant vs control PCR products, and purified PCR products were analysed by Sanger sequencing.

### Statistics

The null hypotheses that near-splice and branchpoint MAPS scores did not significantly differ from synonymous variants were tested with two-sided chi-squared tests of the observed vs the expected number of singletons in each variant class. In order that the synonymous MAPS did not equal zero, all MAPS scores were first corrected by the addition of the synonymous unadjusted proportion of singletons. For each variant class, the “observed” proportion of singletons was taken as the number of alleles multiplied by the corrected MAPS score for that variant class. The “expected” number of singletons was taken as the number of alleles multiplied by the corrected MAPS score for synonymous variants. Multiple testing was accounted for by Bonferroni correction: 79 tests at alpha = 0.05 gave a significance threshold of < 6.3 × 10^−4^.

## Results

### Signals of constraint at near-splice positions are replicated in a large healthy cohort

To estimate the deleteriousness of variation in near-splice positions, we calculated aggregate measures of evolutionary conservation, selective constraint, and predicted splicing disruption for nucleotides within near-splice regions genome-wide.

**Fig. 1:**
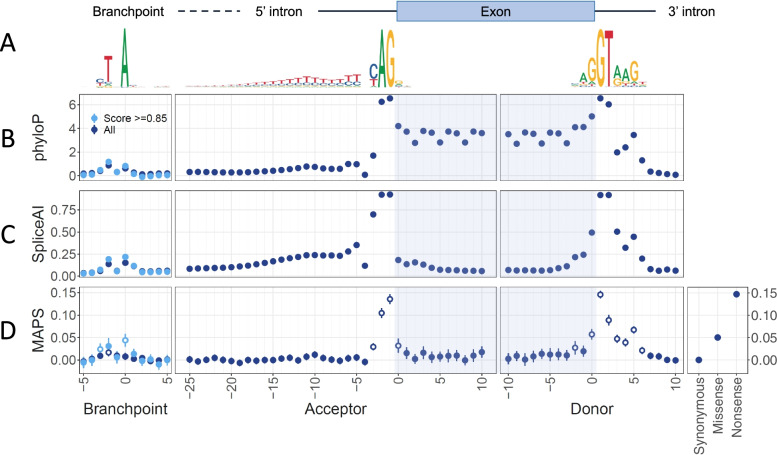
Conservation, predicted splice disruption, and constraint at near-splice and branchpoint positions across 207,548 CDS features in protein-coding genes. **A** Sequence logos and schematic indicating the position of conserved splicing motifs relative to exon/intron boundaries. Positional weight matrices were derived from the human reference sequence at our positions of interest (defined in the “Methods” section). **B** The mean phyloP 100-way scores at splicing positions. Error bars indicate 95% confidence intervals. **C** SpliceAI scores for all possible near splice SNVs. Scores represent the mean probability that any variant at this position disrupts splicing, as predicted by SpliceAI (see the “Methods” section). Error bars represent the 95% confidence interval. **D** Mutability-adjusted proportion of singletons (MAPS) for both coding and near-splice SNVs. Error bars indicate 95% confidence intervals. Positions with a significantly higher MAPS than synonymous variants are indicated with open circles (see the “Methods” section). For branchpoint positions, dark blue points represent all putative branchpoints, whereas light blue points represent the branchpoints with a LaBranchoR score > 0.85

Evolutionary conservation was measured by base-wise phyloP score. The CSSs are very highly conserved (mean phyloP = 6.34) (Fig. 1). Other intronic splicing positions with high phyloP scores include the D + 5 (3.44), D + 4 (2.39), D + 3 (1.97), A-3 (1.70), and D + 6 (1.29) sites. Notably, the A-4 position is very weakly conserved (0.076). Coding positions are generally more highly conserved than intronic sequences. The redundancy of third codon positions and bias for in-phase exons [38] is reflected in lower phyloP scores at every third position, except for the donor 0 position (mean phyloP = 5.01), which is more highly conserved than any other coding position.

To measure selective constraint at near-splice positions, we calculated the degree of purifying selection acting at near-splice positions using MAPS [13]. MAPS was calculated across near splice positions genome-wide, using every observed synonymous, missense, nonsense, and near-splice SNV in 207,548 distinct CDS exons for 26,660 unaffected parents in the 100KGP Rare Disease cohort. The most significant signals of purifying selection are at the CSS, with MAPS scores of 0.089–0.146 (*p* < 1.2 ×  × 10^−43^), approaching those of nonsense variants (0.16) (Fig. 1). The non-canonical positions with a MAPS score significantly above the synonymous baseline after Bonferroni correction include the D-2, D0, D + 3, D + 4, D + 5, D + 6, A-3, and A + 1 positions (*p* < 6.3 × 10^−4^). The MAPS scores at D0 and D + 5 variants (MAPS = 0.057 and 0.067, respectively) are comparable to that at missense variants (0.052). These results are highly concordant with previous near-splice MAPS calculations in the Deciphering Developmental Disorders study (DDD) and ExAC datasets [8].

### A subset of splicing branchpoints are highly constrained

Having replicated earlier findings [8] in our cohort, we expanded our analysis to examine splicing branchpoints, which have not been previously characterised using MAPS.

We repeated our analysis of conservation, constraint, and SpliceAI predicted splicing disruption using a set of 195,863 putative branchpoints predicted by LaBranchoR [31], a deep-learning tool trained on experimentally validated branchpoints.

Annotating each position with base-wise phyloP scores, we found modest conservation of BP0 (0.62) and BP-2 (0.87), consistent with previous results [31, 39, 40] (Fig. 1).

Next, we calculated the MAPS statistic for these branchpoint positions in the same cohort described above. When all putative branchpoints were considered, only the BP-2 position has a significantly higher MAPS score than the synonymous baseline (MAPS = 0.017, *p* = 1.3 × 10^−4^) (Fig. 1). However, when only the most confident branchpoints are considered (LaBranchoR score > 0.85, *n* = 57,342), the BP0 (MAPS = 0.044, *p* = 7.6 × 10^−9^) and BP-3 (0.024, *p* = 3.7 × 10^−4^) are also significantly constrained, with nominal constraint at BP-2 (MAPS = 0.031, *p* = 1.0 × 10^−3^). We further stratified the MAPS analysis by reference allele at the BP-1 and BP-3 positions but were generally underpowered to detect motif-specific constraints (Additional file 1: Fig. S1). These data suggest that LaBranchoR-predicted branchpoints are functionally important and that variants near branchpoints may be a significant cause of rare disease.

Next, we calculated SpliceAI scores for every possible SNV around each branchpoint. Again, variants at BP0 (mean SpliceAI = 0.15) and BP-2 (mean SpliceAI = 0.14) are nominally more likely to disrupt splicing than synonymous coding variants (Fig. 1). This trend is more pronounced when only the most confident branchpoints (LaBranchoR score > 0.85, *n* = 57,342) are considered (mean SpliceAI BP0 = 0.22, BP-2 = 0.19).

### New diagnostic candidates among near-splice de novo variants

Having described three orthogonal metrics which independently suggest that certain near-splice and branchpoint variants may be deleterious, we sought to identify new candidate diagnostic variants at these positions.

**Fig. 2:**
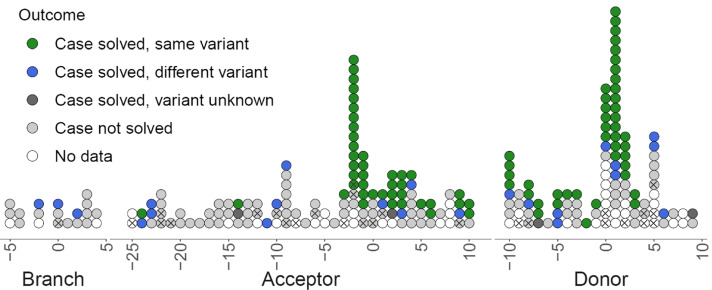
Participant outcomes for rare disease probands with de novo splicing variants in known monoallelic loss-of-function rare disease genes. Each point represents a DNV in a rare disease proband. Points are coloured by the clinical outcome for that individual. Crosses indicate variants which were identified as likely new diagnoses in this study. Where a variant overlaps both a branchpoint and a splice acceptor position, only the splice acceptor annotation is given

We interrogated a set of 870,559 DNVs in 12,028 trios for potentially diagnostic splicing variants. We identified 258 de novo SNVs overlapping near-splice or branchpoint regions of coding exons in known monoallelic “loss of function” rare disease genes in 255 individuals (Additional file 2: Table S1). Of these, 238 were in near-splice positions (spanning intronic and exonic positions), and 20 were within 5 bp of a putative branchpoint (Fig. 2) (12 variants had both a splice acceptor and a branchpoint annotation; in these cases, only the splice acceptor annotation (e.g. A-25) was kept). To maximise our sensitivity to identify new candidate diagnoses, variants were prioritised only by their near-splice location, without additional filtering by, for example, SpliceAI score.

Reviewing tiering data from the 100KGP bioinformatics pipeline, we found that of these 258 variants, 83 (32%) were “tier 1”, 46 (18%) were “tier 3”, and 129 (50%) were not tiered (Additional file 1: Fig. S2). Of 59 CSS variants, 36 (61%) were “tier 1”, nine (15%) were “tier 3”, and 14 (24%) were not tiered. Of ten donor + 5 variants, four were “tier 1”, two were “tier 3”, and four were not tiered (Additional file 1: Fig. S2). Annotation of these variants with SpliceAI generally highlighted variants at positions with high MAPS scores (Additional file 1: Fig. S3).

A total of 212 participants with a near-splice DNV had outcome data in the form of “exit questionnaires” from their referring Genomic Medicine Centre. In 84/111 (76%) of solved cases, the diagnostic variant matched our near-splice finding (Fig. 2). This result gives us confidence in our approach to candidate variant identification. Nevertheless, a significant proportion of participants with completed exit questionnaires had unsolved cases (101/212, 48%). These included nine with a DNV in the CSS of a known rare disease gene, one with a donor 0 variant, and four with donor + 5 variants, which have previously been estimated to have a 90% positive predictive value in rare disease diagnosis [8] (Fig. 2).

For each participant and DNV, we manually reviewed the similarity between the HPO terms recorded at recruitment and the phenotype expected for a loss-of-function variant in that gene. Excluding any participants whose case was already solved through 100KGP, we identified 35 new likely diagnostic variants with at least a plausible phenotype match (Additional file 3: Table S2). We placed a clinical collaboration request with Genomics England in each case, to recruit the participant for functional characterisation of the variant with RNA studies.

### New diagnoses among the cohort

Whole blood RNA samples were obtained for five participants with near splice DNVs. RT-PCR was used to characterise the splicing impact of each variant (Additional file 1: Fig. S4). Abnormal splicing events (all exon skipping) were detected in four participants (participants 74 (*ARID1A*, A-3), 249 (*USP7*, D + 5), 259 (*TLK2*, D + 5), 261 (*KAT6B*, D + 5)). In the remaining participant (participant 32 (*PPP1R12A*, A-21)), no disruption to splicing was observed (Table 1, Additional file 1: Fig. S4). Notably, these functional outcomes are consistent with the SpliceAI score for the variant in each case (Table 1). For two additional participants where the candidate variant fell in a canonical splice site (participants 83 (*TAOK1*, A-2) and 94 (*PHIP*, A-2)), a new diagnosis was reached without the need for functional work based on ACMG criteria with a PVS1 classification for these variants (Table 1, Additional file 1: Fig. S4).

**Table 1:** Diagnostic outcomes for seven individuals after clinical and functional characterisation of the splicing variant. Five individuals underwent RNA studies, of which four received a new diagnosis. In two additional individuals, a diagnosis was reached without the need for RNA studies. In total, a new diagnosis was confirmed for six individuals. Note that the given HPO terms are “abstracted” (see the “Methods” section) to protect confidentiality. *In participants 83 and 94, a new diagnosis was reached without the need for functional evaluation. **This exit questionnaire outcome was updated after the participant was identified by this study. DS_any: the probability that the variant has any impact on splicing (see the “Methods” section). DS_max: the maximum SpliceAI delta score of the variant. DS_max_type: the predicted splicing impact with the maximum delta score (DS_DL = donor loss, DS_DG = donor gain, DS_AL = acceptor loss, DS_AG = acceptor gain)

ID	Chrom	Pos	Ref	Alt	Region	Site	Symbol	ENST	DS_any	DS_max	DS_max type	Tier	Max tier	Exit questionnaire	HPO terms (abstracted)	Splicing impact	Outcome
74	chr1	26767787	C	G	acceptor	-3	ARID1A	ENST00000324856	0.71	0.65	DS_AL	3	3	No data	Aplasia/Hypoplasia of the mandible, Advanced eruption of teeth, Abnormal pulmonary valve morphology, Abnormality of calvarial morphology, Abnormality of cardiovascular system morphology, Oral cleft	Exon skipping	New diagnosis
261	chr10	74989117	G	A	donor	+5	KAT6B	ENST00000287239	0.98	0.98	DS_DL	3	3	Case not solved	Hypothyroidism	Exon skipping	New diagnosis
259	chr17	62596679	G	A	donor	+5	TLK2	ENST00000326270	0.97	0.96	DS_DL	3	3	Case not solved	Abnormality of globe location, Abnormal facial shape, Facial asymmetry, Abnormal heart sound, Cutaneous syndactyly, Abnormal ear morphology, Neurodevelopmental delay, Short stature, Abnormal digit morphology, Decreased body weight, Intrauterine growth retardation, Abnormality of higher mental function, Motor delay, Language impairment, Gait disturbance, Abnormal location of ears	Exon skipping	New diagnosis
249	chr16	8905182	C	A	donor	+5	USP7	ENST00000344836	0.932	0.8	DS_DL	3	3	Case not solved	Motor delay, Abnormal size of the palpebral fissure, Abnormal hair quantity, Abnormality of globe location, Facial hypertrichosis, Neurological speech impairment, Abnormality of higher mental function, Abnormal metatarsal morphology	Exon skipping	New diagnosis
94	chr6	79002126	T	G	acceptor	-2	PHIP	ENST00000275034	1	1	DS_AL	3	2	Case not solved	Abnormality of higher mental function, Motor delay, Neurodevelopmental delay, Macrotia, Language impairment, Finger clinodactyly, Abnormal muscle tone, Facial hypertrichosis	N/A	New diagnosis*
83	chr17	29491782	A	G	acceptor	-2	TAOK1	ENST00000261716	0.992	0.99	DS_AL	3	3	Case solved, same variant**	Renal agenesis, Hemangioma, Abnormality of joint mobility, Hypotonia, Increased head circumference, Neurodevelopmental delay	N/A	New diagnosis*
32	chr12	79808598	T	A	acceptor	-21	PPP1R12A	ENST00000450142	0	0	DS_AG	N/A	3	Case not solved	Increased head circumference, Abnormal thorax morphology, Bowing of the legs, Growth delay, Limb undergrowth, Abnormality of joint mobility, Short digit, Neurodevelopmental abnormality, Abnormality of movement	Normal splicing	Unsolved

In summary, we demonstrate a functional splicing defect in four out of five participants recruited to our study, and we have identified a new molecular diagnosis for six individuals to date.

## Discussion

We examined WGS data from 38,688 individuals in the Rare Disease arm of the 100KGP to evaluate the contribution of splicing variants to rare genetic diseases. Using a population-based metric of constraint, MAPS, we showed that certain near-splice and branchpoint positions are under strong purifying selection, consistent with previous work [8, 40, 41]. We identified 258 de novo near-splice and branchpoint variants in known disease genes in these families. We identified 35 likely diagnostic variants which had previously been missed through the 100KGP, and we have confirmed a new molecular diagnosis for six participants to date. Overall, we demonstrate the clinical value of examining both canonical and non-canonical splicing variants in unsolved rare diseases.

### Non-canonical splicing positions harbour deleterious splicing variants

We used three orthogonal approaches to estimate the deleteriousness of near-splice and branchpoint variants: between-species conservation, within-species constraint, and predicted splicing disruption. The PhyloP, MAPS, and SpliceAI scores at splicing positions consistently highlight those non-canonical splicing positions (especially D0 and D + 5) which are likely to harbour damaging variants. Indeed, three out of three D + 5 variants in which we performed RNA studies caused exon skipping. Importantly, although we use a cohort of unaffected parents as a proxy for a normal population, the MAPS data we present is highly concordant with the strong signals of negative selection at which have been previously described in the ExAC and DDD datasets [8].

Extending this analysis to splicing branchpoints, we find strong signals of negative selection at a subset of branchpoint positions. These results are consistent with other measures of constraint previously described at bovine and human branchpoints [41]. We also identified candidate diagnostic variants at these positions, including several overlapping experimentally validated branchpoint positions (Additional file 4: Table S3), and we are awaiting RNA samples to functionally characterise these variants. The disruption of splicing branchpoints may therefore make an important contribution to rare disease [11, 12]. A systematic analysis of de novo variation at putative branchpoints and a comparison of the utility of different branchpoint prediction tools in a clinical setting are exciting future research opportunities.

The ACMG variant interpretation guidelines give special status to CSS variants as “very strong” diagnostic candidates in disorders where LoF is a known disease mechanism [6]. This remains the case in more detailed guidance for the interpretation of LoF variants which has recently been introduced [42]. However, the deleteriousness of splicing variants is not binary, but on a continuum, and can be quantitatively compared to other variant classes. Previous estimates suggest that 46% of non-canonical near-splice DNVs in dominant rare disease genes may be pathogenic, rising to 71% for pyrimidine to purine transversions in the polypyrimidine tract, and 90% for D + 5 variants [8]. The deleteriousness of individual variants is contingent on many factors, such as local sequence context, the alternate nucleotide, exon frame, exon length, and intron length [9]. For this reason, the systematic classification of near-splice variants remains challenging, and clinical interpretation of these variants is still dependent on expert phenotype matching and functional validation of candidate variants.

The functional characterisation of splicing variants can be challenging and requires adequate amounts of good-quality RNA. Our study is limited by the use of blood as a proxy for the most clinically relevant tissue, although we affirm the utility of blood RNA analysis by identifying splicing defects in four out of five samples tested. Whereas RT-PCR is a bespoke and low-throughput approach, going forward, RNA-sequencing (RNA-seq) offers an unbiased and high-throughput alternative to simultaneously detect and functionally characterise splicing variants. A whole-transcriptome RNA-seq pilot study has recently been proposed for 100KGP, and the use of RNA-seq in routine clinical practice could offer a much-needed means to systematically and objectively interpret splicing variants [43].

### New rare disease diagnoses

We identified 258 de novo SNVs overlapping near-splice or branchpoint regions of known monoallelic “loss of function” rare disease genes in 255 individuals. Of these, at least 84 were already considered to be diagnostic through 100KGP, and we identified an additional 35 variants which are likely to be diagnostic given the available molecular, phenotypic, and in silico data. We confirmed a new molecular diagnosis for six participants, including four participants for whom RNA studies were performed.

Surprisingly, several strong diagnostic candidates were apparently overlooked in the standard variant interpretation pipeline, including at least nine CSS variants and four D + 5 variants, all in known rare disease genes. Of ten de novo D + 5 variants, none were previously labelled as pathogenic, despite their high prior probability of being diagnostic in this context [8].

Clearly, many new diagnoses remain to be found. A recent analysis of 100KGP data in the context of craniosynostosis found that expert-led review more than doubled diagnostic yields compared to the standard pipeline [3]. An important factor is that the “virtual panels” applied to variant calls are outdated and do not include recently discovered disease genes. Notably, the majority of new likely diagnostic variants we identify are intronic (26/35), likely because these variants are excluded from the 100KGP tiering and prioritisation pipeline and are therefore not subjected to detailed clinical interpretation. Our phenotype-matching work suggests that the clinical impact of near-splice variants has been under-ascertained in this cohort, and we are continuing to recruit participants for functional assessment of these variants. Additionally, the analysis of more distal variants overlapping exonic splicing enhancers and silencers or cryptic splice sites in deep-intronic regions offers an important future research opportunity.

One obstacle to increasing the number of researcher-identified diagnoses in this context is the difficulty of recontacting de-identified participants and clinicians through secure research environments. The confidentiality of all participants in research is rightly a priority, and new pathways must be developed to streamline the clinical-research interface in medical genomics.

## Conclusions

In conclusion, the disruption of splicing is an important cause of rare diseases among 100KGP participants, but the contribution of non-canonical variants is still under-recognised. Splicing branchpoints are another non-canonical and non-coding source of damaging splicing variants which are amenable to systematic analysis in WGS data. The improved interpretation of splicing variants is an area of great promise to genomic medicine and, above all, to individuals with rare diseases and their families.

## Supplementary Information

**Additional file 1.** Supplementary Figs. S1, S2, S3, S4.

**Additional file 2.** Table S1. Prioritised variants.

**Additional file 3. **Table S2. Candidate variants.

**Additional file 4. **Table S3. Experimentally validated branchpoints.

## Data Availability

All analyses were performed within a protected research environment which is available to registered users (see https://www.genomicsengland.co.uk/about-gecip/for-gecip-members/data-and-data-access/). All data used in this study are available within this research environment. Specifically, clinical data and tiering data were accessed through the LabKey application within this research environment. Controlled access to this data is limited to protect the privacy and confidentiality of participants in the Genomics England 100,000 Genomes Project and to comply with the consent given by those participants for use of their healthcare and genomic data. Access to these data is permitted to bona fide researchers after registration with a Genomics England Clinical Interpretation Partnership (GeCIP, see link above). Applications to join a GeCIP are reviewed within 10 working days. This project was registered with Genomics England under Research Registry ID RR143 and RR166. Extended functional data generated in this study are available in the supplementary materials. Extended clinical and participant information, within the limits of participant consent and data access agreements with Genomics England, is available in the supplementary materials. The analyses were performed using Python 3.7.6 and Pandas 1.2.1. The figures were generated in R 3.5.2 using RStudio 1.1.463. All code is available online at https://github.com/alexblakes/100KGP_splicing20.
