# Supplementary material for: A systematic analysis of splicing variants identifies new diagnoses in the 100,000 Genomes Project
Source: Genome Med. 2022 Jul 26;14:79. doi: 10.1186/s13073-022-01087-x (PMC9327385; doi:10.1186/s13073-022-01087-x)
Supplement: Supplementary file 1 — Additional file 1. Supplementary Figs. S1, S2, S3, S4. [file 13073_2022_1087_MOESM1_ESM.pdf]

Fig S1

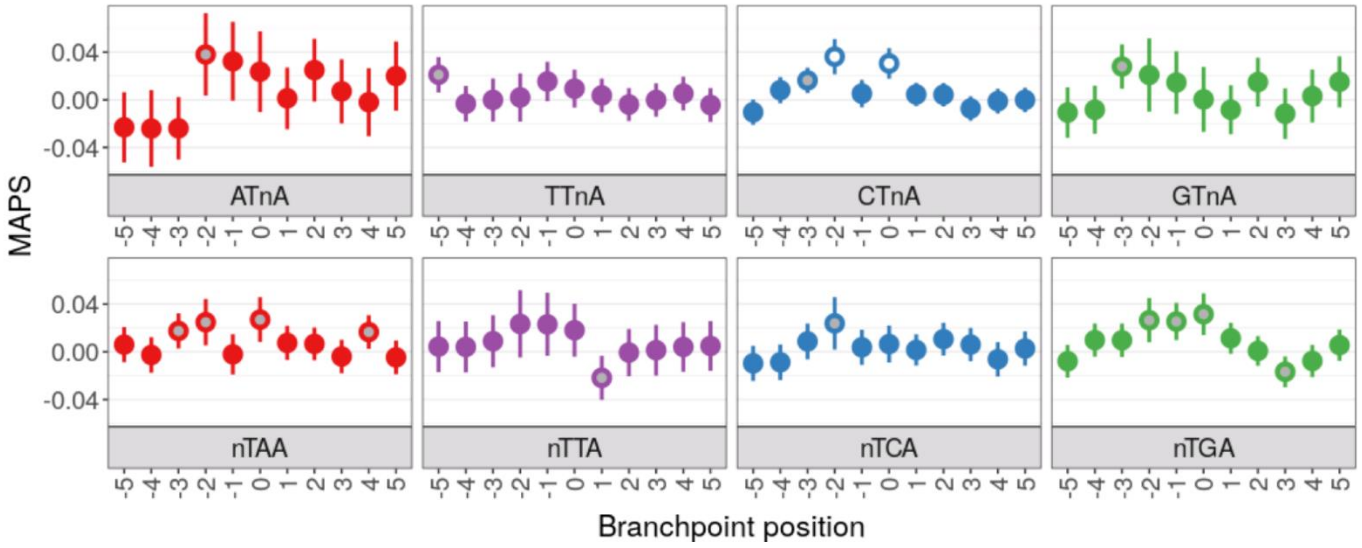

Constraint at predicted splicing branchpoints, stratified by reference allele at BP-3 and BP-1 positions. For LaBranchoR-predicted branchpoints with reference A and T nucleotides at the BP0 and BP-2 positions (nTnA motif, n = 165,564), we calculated MAPS after stratifying by the reference allele at BP-3 and BP-1 positions. Sites which are significantly constrained after Bonferroni correction (88 tests, alpha = 0.05, $p < 5.8 \times 10^{-4}$) are shown with white points. Sites with nominally significant constraint ($p < 0.05$) are shown with grey points. We are generally underpowered to detect motif-specific constraints at these loci.

Fig S2

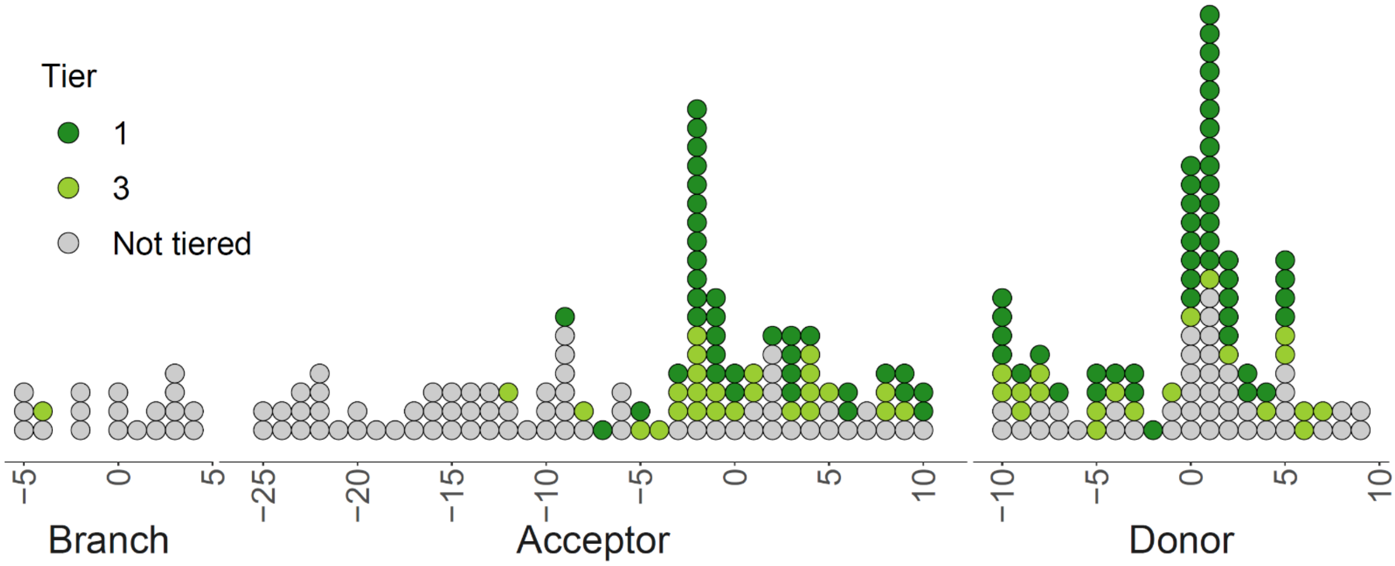

Tiering data for splicing DNVs in known monoallelic loss-of-function rare disease genes. Each point represents a DNV in a rare disease proband. Points are coloured by the "tier" of that variant in the GEL annotation pipeline (see Methods).

Fig S3

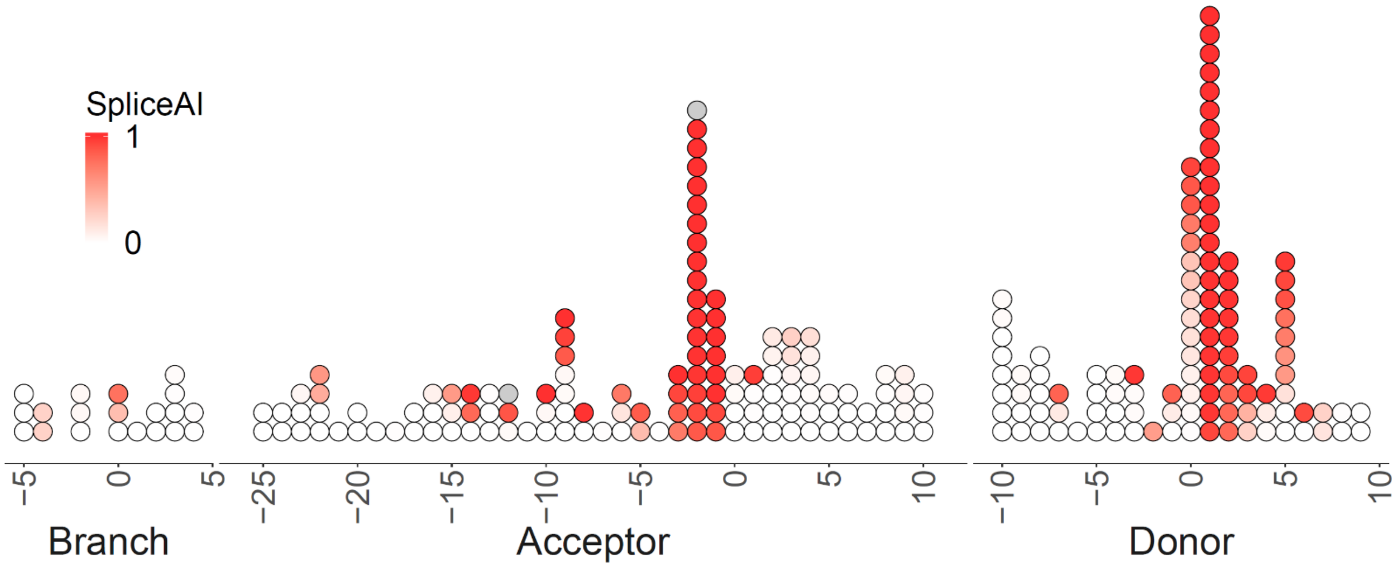

SpliceAI scores of splicing DNVs in known monoallelic loss-of-function rare disease genes. Each point represents a DNV in a rare disease proband. Points are coloured by SpliceAI score; grey points indicate that no SpliceAI annotation is available.

Fig S4

<table>
<tr><td>Variant, gene</td><td colspan="2">chr10:74989117 G>A, KAT6B</td></tr>
<tr><td>Sequence schematic</td><td colspan="2">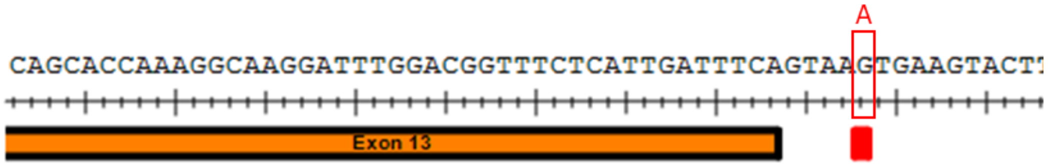
</td></tr>
<tr><td>Splicing schematic</td><td colspan="2">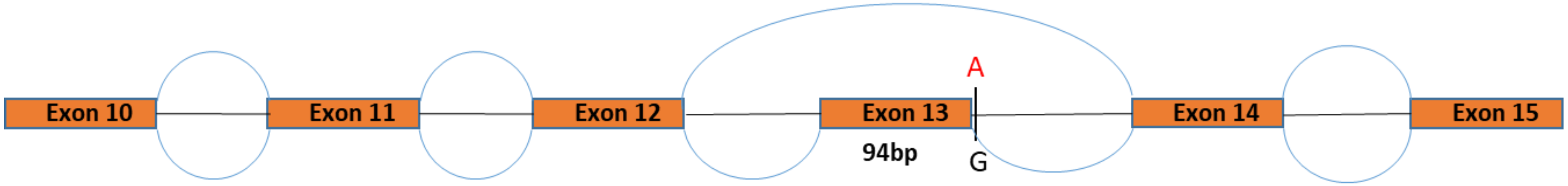
</td></tr>
<tr><td>Gel electrophoresis</td><td>Annotated gel image
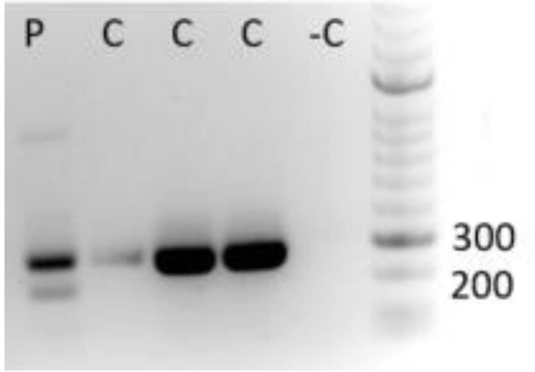

P=Proband, C=Control, -C=DNA free control</td><td>Full gel image
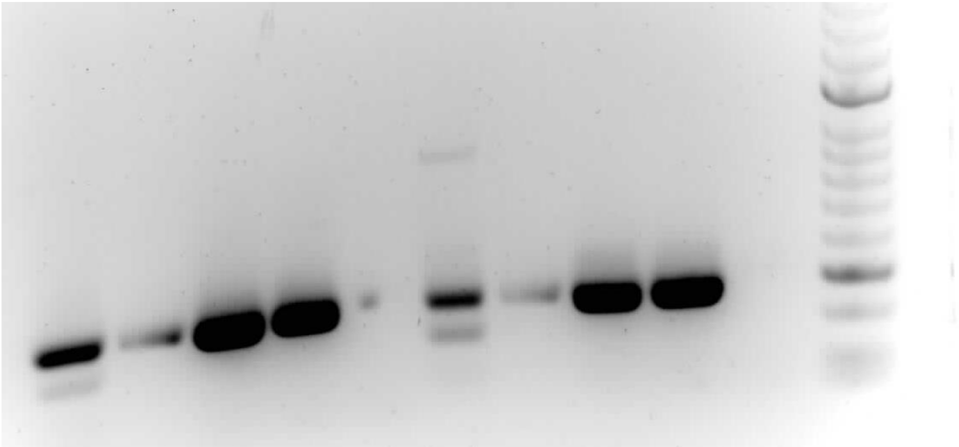</td></tr>
<tr><td>Sanger sequencing proband specific band</td><td colspan="2">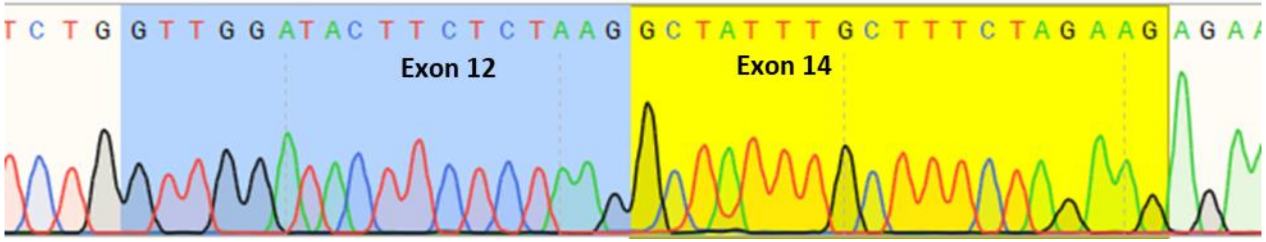

Exon 13 skipped</td></tr>
</table>

Variant, gene	chr17:62596679 G>A, *TLK2*	
Sequence schematic	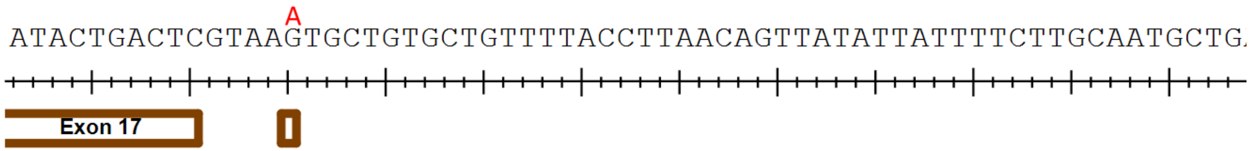	
Splicing schematic	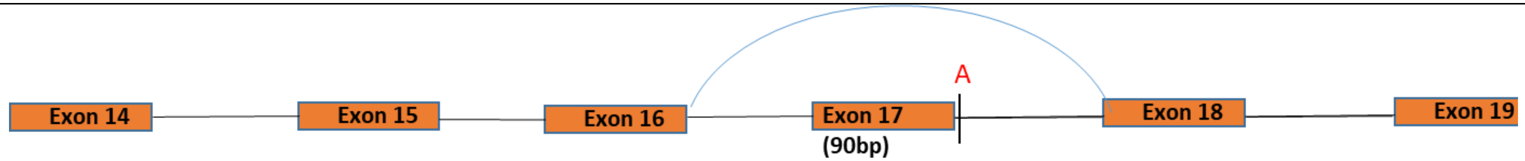	
Gel electrophoresis	Annotated gel image<br>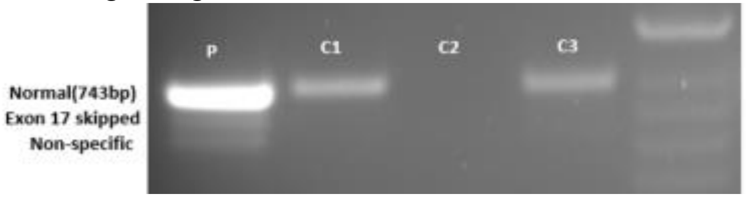<br>P=Proband, C=Control	Full gel image<br>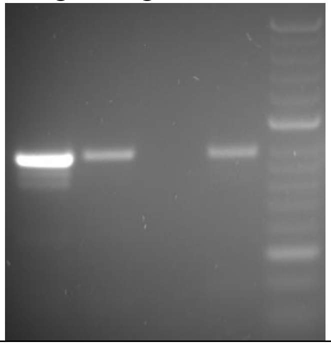
Sanger sequencing proband specific band	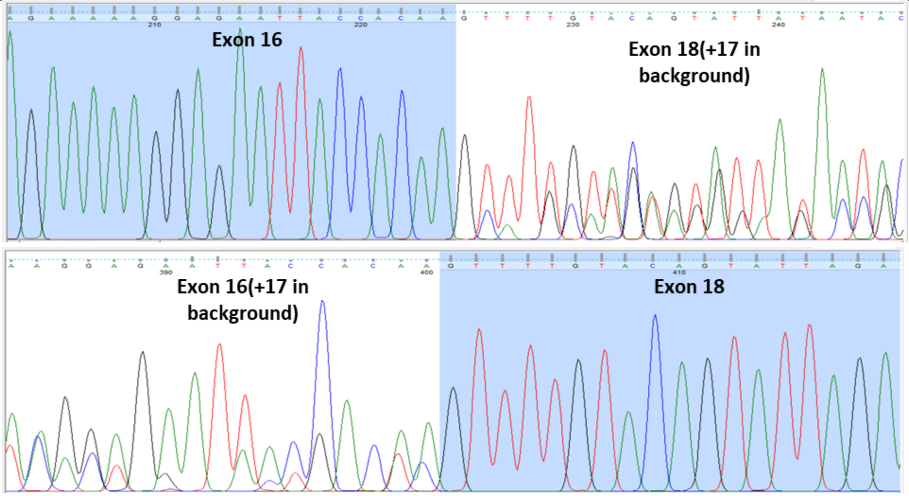<br>Exon 17 skipped	

Variant, gene	chr1:26767787 C>G, *ARID1A*
Sequence schematic	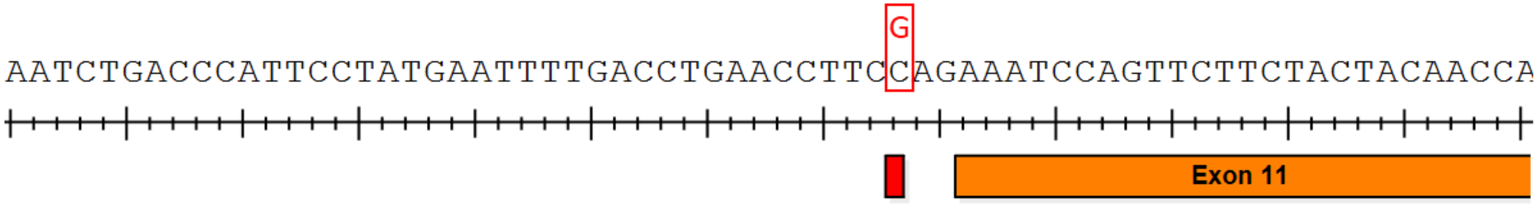
Splicing schematic	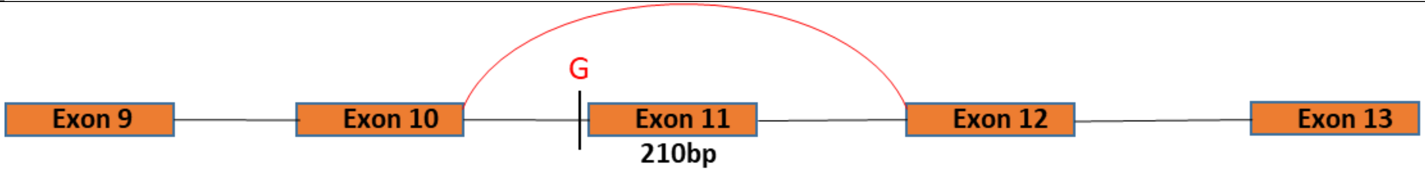
Gel electrophoresis	Annotated gel image<br>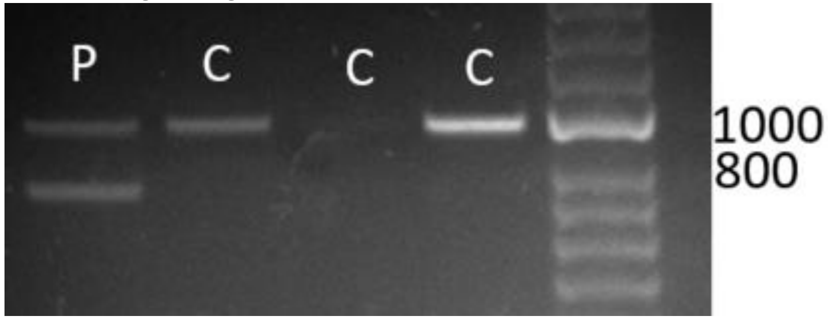<br>P=proband, C=Control<br><br>Full gel image<br>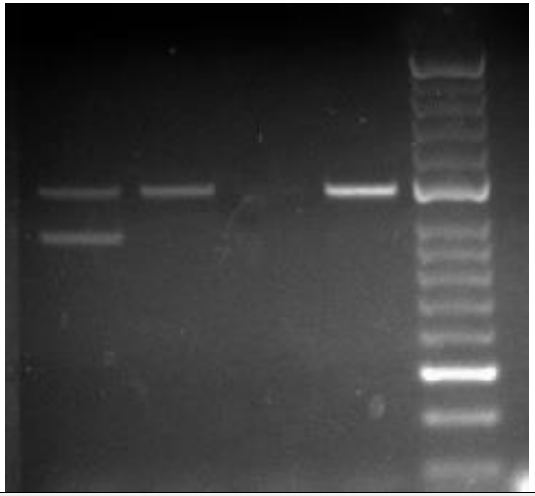
Sanger sequencing proband specific band	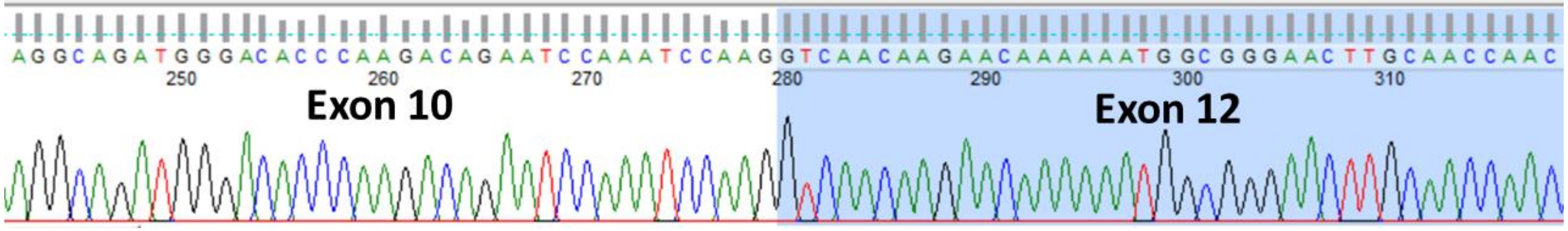<br>Exon 11 skipped

Variant, gene	chr16:8905182 C>A, *USP7*
Sequence schematic	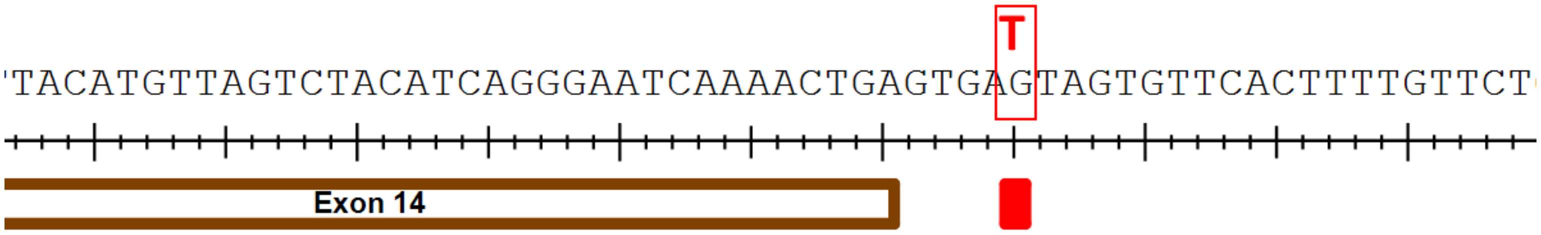
Splicing schematic	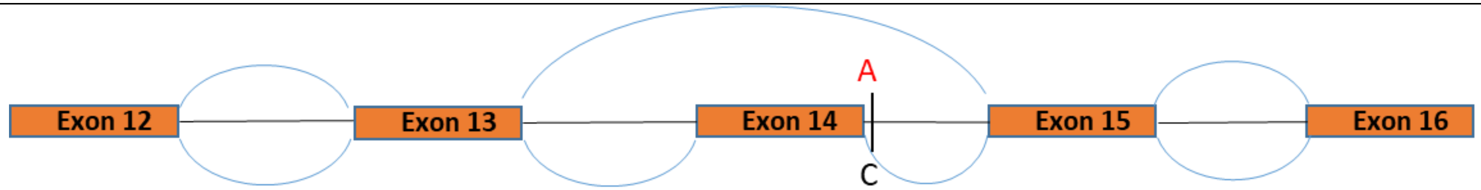
Gel electrophoresis	Annotated gel image<br>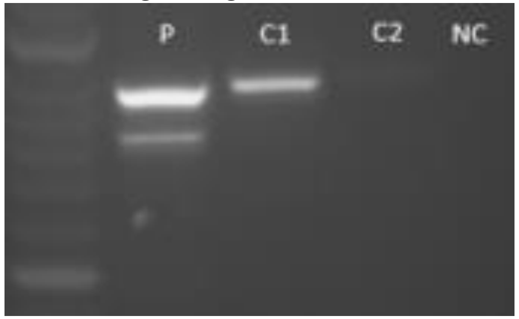<br>P=proband, C=Control, NC = no template control<br>Full gel image<br>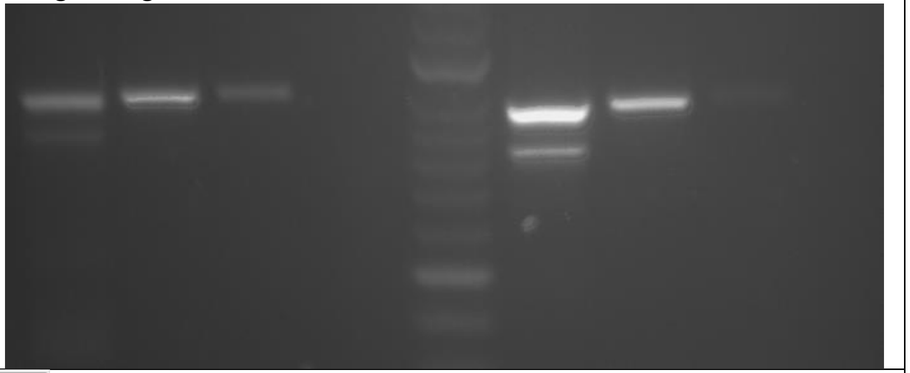
Sanger sequencing proband specific band	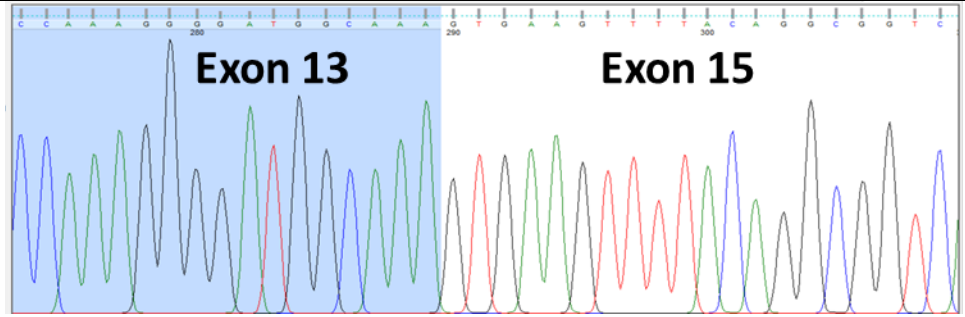<br>Exon 14 skipped

<table>
<tr><td>Variant, gene</td><td colspan="2">chr12:79808598 T>A, PPP1R12A</td></tr>
<tr><td>Sequence schematic</td><td colspan="2">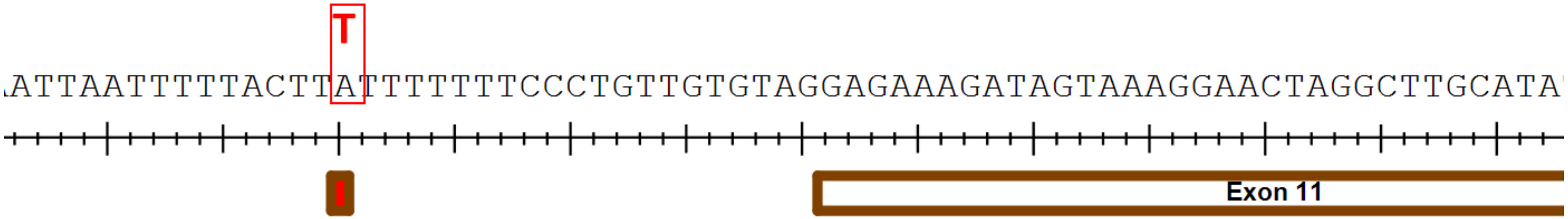
</td></tr>
<tr><td>Splicing schematic</td><td colspan="2">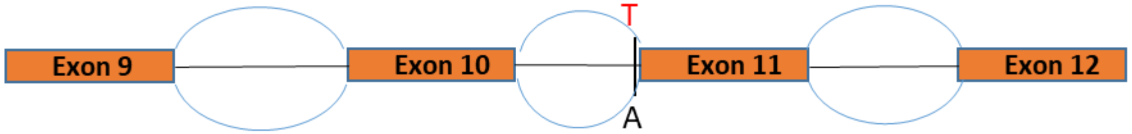
</td></tr>
<tr><td>Gel electrophoresis</td><td>Annotated gel image
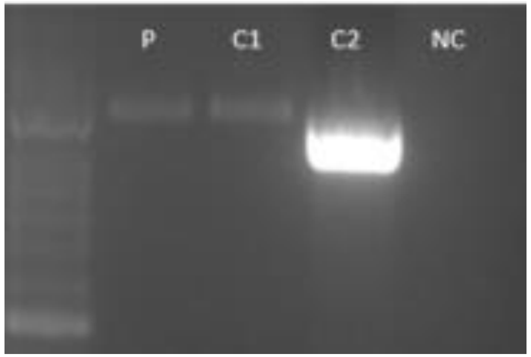

P=proband, C=Control, NC = no template control</td><td>Full gel image
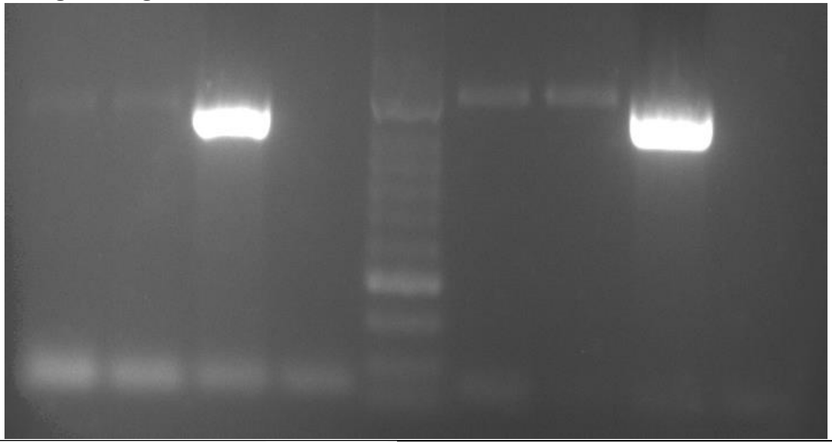</td></tr>
<tr><td>Sanger sequencing</td><td colspan="2">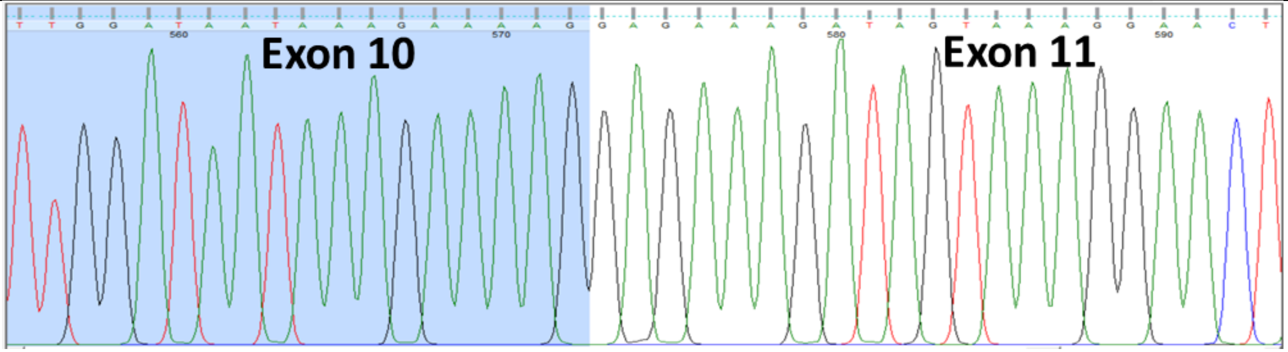

Normal splicing</td></tr>
</table>

Functional outcomes for participant samples which were characterised by RT-PCR. Each page illustrates the following: a schematic of variant position relative to the exon/intron junction, a schematic of the splicing consequence of each variant, gel electrophoresis of amplified RT-PCR products for participant and control samples (cropped annotated image for clarity, plus full gel image), Sanger sequencing trace for proband-specific bands.
